# Changes in health facility readiness for obstetric and neonatal care services in Nepal: an analysis of cross-sectional health facility survey data in 2015 and 2021

**DOI:** 10.1186/s12884-023-06138-8

**Published:** 2024-01-24

**Authors:** Sabita Tuladhar, Deepak Paudel, Eva Rehfuess, Matthias Siebeck, Cornelia Oberhauser, Maria Delius

**Affiliations:** 1 Teaching & Training Unit, Division of Infectious Diseases and Tropical Medicine, University Hospital, LMU, Munich, Germany; 2 Center for International Health, LMU, Munich, Germany; 3 Save the Children, Kathmandu, Nepal; 4 Institute for Medical Information Processing, Biometry and Epidemiology, LMU, Munich, Germany; 5 Pettenkofer School of Public Health, Munich, Germany; 6 Institute of Medical Education, LMU, University Hospital, LMU, Munich, Germany; 7 Department of Obstetrics and Gynecology, University Hospital, LMU, Munich, Germany

**Keywords:** Delivery services, Maternal health, Newborn health, Quality of care, Emergency obstetric and neonatal care

## Abstract

**Background:**

Nepal is committed to achieving the Sustainable Development Goal (SDG) 2030 target 3.1 of reducing the maternal mortality ratio to 70 deaths per 100,000 live births. Along with increasing access to health facility (HF)-based delivery services, improving HF readiness is critically important. The majority of births in Nepal are normal low-risk births and most of them take place in public HFs, as does the majority of maternal deaths. This study aims to assess changes in HF readiness in Nepal between 2015 and 2021, notably, if HF readiness for providing high-quality services for normal low-risk deliveries improved; if the functionality of basic emergency obstetric and neonatal care (BEmONC) services increased; and if infection prevention and control improved.

**Methods:**

Cross-sectional data from two nationally representative HF-based surveys in 2015 and 2021 were analyzed. This included 457 HFs in 2015 and 804 HFs in 2021, providing normal low-risk delivery services. Indices for HF readiness for normal low-risk delivery services, BEmONC service functionality, and infection prevention and control were computed. Independent sample T-test was used to measure changes over time. The results were stratified by public versus private HFs.

**Results:**

Despite a statistically significant increase in the overall HF readiness index for normal low-risk delivery services, from 37.9% in 2015 to 43.7%, in 2021, HF readiness in 2021 remained inadequate. The availability of trained providers, essential medicines for mothers, and basic equipment and supplies was high, while that of essential medicines for newborns was moderate; availability of delivery care guidelines was low. BEmONC service functionality did not improve and remained below five percent facility coverage at both time points. In private HFs, readiness for good quality obstetrical care was higher than in public HFs at both time points. The infection prevention and control index improved over time; however, facility coverage in 2021 remained below ten percent.

**Conclusions:**

The slow progress and sub-optimal readiness for normal, low-risk deliveries and infection prevention and control, along with declining and low BEmONC service functionality in 2021 is reflective of poor quality of care and provides some proximate explanation for the moderately high maternal mortality and the stagnation of neonatal mortality in Nepal. To reach the SDG 2030 target of reducing maternal deaths, Nepal must hasten its efforts to strengthen supply chain systems to enhance the availability and utilization of essential medicines, equipment, and supplies, along with guidelines, to bolster the human resource capacity, and to implement mechanisms to monitor quality of care. In general, the capacity of local governments to deliver basic healthcare services needs to be increased.

**Supplementary Information:**

The online version contains supplementary material available at 10.1186/s12884-023-06138-8.

## Introduction

Nepal made good progress in reducing the maternal mortality ratio (MMR) over the last two and half decades, as illustrated by a 72.2% MMR reduction, from 539 to 151 per 100,000 live births between 1996 and 2021 [1, 2]. However, the rate of decline in MMR has not been uniform over time: between 1996 and 2016, Nepal observed modest gains in maternal survival with a 2.6% annual MMR reduction; in contrast, after 2016 until 2021 the MMR reduction accelerated to an annual rate of decline of 6.9% [1, 2]. Because of the slow rates of progress until 2016, Nepal’s endeavor to meet the United Nations (UN) Millennium Development Goal (MDG) 4 of reducing the MMR to 134 per 100,000 live births by 2015 [3] was only partly realized. The challenge continues with regard to reaching the ambitious UN Sustainable Development Goal (SDG) target 3.1 of reducing the MMR to less than 70 per 100,000 live births by 2030 [4], which calls for a further 53.6% reduction of the MMR from the levels observed in 2021, translating into a 5.9% annual rate of reduction. The biggest gains in maternal survival can be achieved through universal access to health facility (HF)-based delivery and immediate postpartum services [5]. Although access to HF-based delivery services has improved in Nepal over the past two decades through a variety of dedicated programs and interventions, there are large inequities across different regions and population groups of the country [1, 6]. Furthermore, the neonatal mortality rate (NMR) has stagnated at 21 per 1,000 live births since 2016 and SDG target 3.2 is reducing it to 12 deaths per 1,000 live births by 2030 [4, 6].

**Fig. 1:**
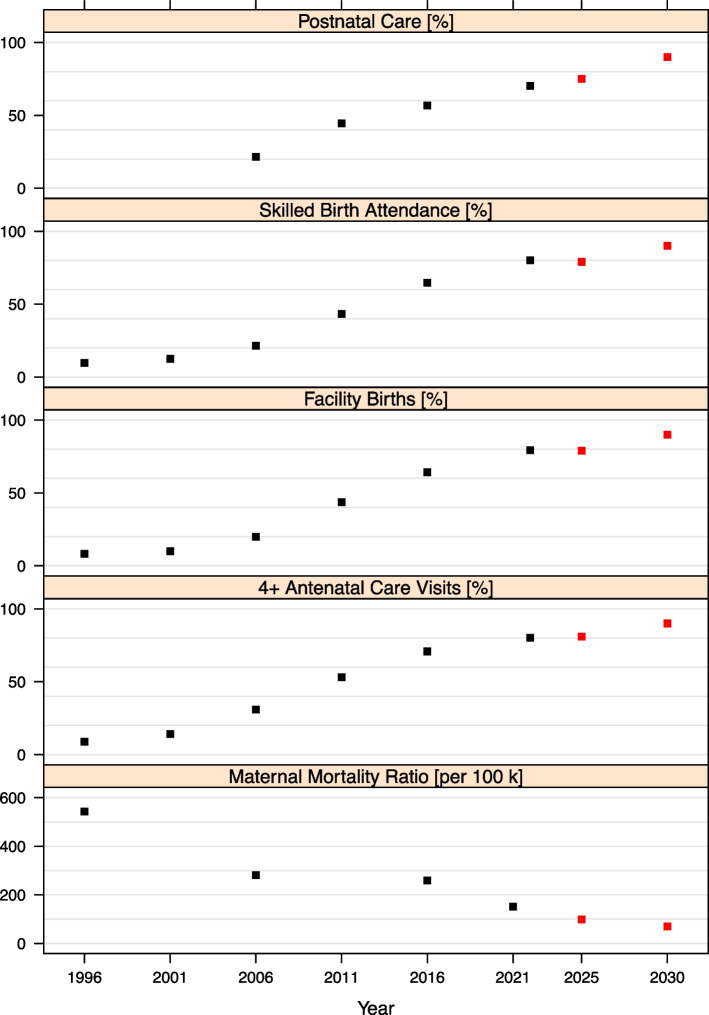
Trends in maternal mortality ratio, antenatal care, health facility births, skilled birth attendance, and postnatal care in Nepal, 1996 to 2022, and SDG targets. Data source: 1996, 2001, 2006, 2011, and 2022: The DHS Program STATCompiler; 2021: Nepal Maternal Mortality Study (NMMS) 2021; 2025 and 2030 targets: SDG Status and Roadmap: 2016-2030

Between 2000 and 2015, the uptake of maternal health services improved steeply in Nepal (Fig. 1). Population coverage with four or more antenatal care visits during the fourth, sixth, eighth, and nine months of pregnancy increased nine-fold from 8.9% in 2001 to 80.2% in 2022, births taking place at a HF changed from a mere 8.2% in 1996 to 79.3% in 2022, and postpartum check ups within two days after delivery improved from 21.5% in 2006 to 70.3% in 2022 [1, 6, 7]. The majority of these services were provided through public HFs, however, over the years, service uptake in private HFs has also been increasing gradually. For example, in 2016, 15.8% of births took place in a private HF compared to only 1.2% of births in 1996 [7].

Normal delivery, as characterised by the World Health Organization (WHO), is spontaneous in onset, low risk at the start of labour, and remains so throughout labour and delivery; the infant is born spontaneously out of a vertex position between 37 and 42 completed weeks of pregnancy; and after birth mother and infant are in good condition [8]. According to the Health Management Information System of Nepal in 2021, 77.8% births were spontaneous, 2.2% assisted, and 20.0% cesarean [9]. In this study, normal spontaneous deliveries are referred to as “normal low-risk deliveries”. To achieve SDG target 3.1, Nepal aims for 90.0% population coverage with regard to recommended antenatal care, births taking place at HFs, and recommended postnatal care by 2030 [4]. Nepal is likely to be on track to meet these targets, provided the current level of investment in the safe motherhood program is continually and sustainably increased. However, for these improvements to be translated into better maternal survival, the increase in access to HF-based delivery services must be matched with improvements in the quality of care (QoC).

QoC is multi-dimensional and implies that health care must be safe, effective, people-centered, timely, efficient, equitable, and integrated [10]. The present need for any country is a high-quality health system that is valued and trusted by all people, can respond to changing population needs and is able to consistently deliver care that improves or maintains health [11]. Currently, the quality of delivery services at HFs is sub-optimal in Nepal, for example, in 2021, only 46.8% of the providers took the woman’s temperature during delivery, and only 48.4% washed their hands properly prior to a physical examination of a woman during delivery [12].

The WHO QoC Framework for Maternal and Newborn Health [13, 14] and the Donabedian QoC Model [15] emphasize three distinct constructs, i.e. *structural factors* (also referred to as facility readiness), *clinical procedures* (comprising the provider’s knowledge, skills, and behavior), and the *patients’ experience of care* as fundamental to ensuring high QoC. Structural factors can be equated with “facility readiness”, i.e. the capacity of HFs to provide high-quality delivery services [16]. Furthermore, HFs offering normal low-risk delivery services should be equipped to provide basic emergency obstetric and neonatal care (BEmONC), which is measured as the seven signal functions and key medical interventions used to treat the direct obstetric complications that cause most maternal deaths around the globe [7].

The recent coronavirus disease (COVID)-19 pandemic posed additional challenges to the delivery of maternal health services and maternal survival in Nepal. The four-month-long COVID-19 lockdown in early-2020 interrupted delivery services in public basic healthcare centers due to facility closure, stock out of medicines, and lack of ambulance services; in contrast, all referral hospitals remained open for delivery, except for the initial days of the lockdown [17]. It also showcased that pregnant and postpartum women and their newborns are at risk of infection in HFs, potentially leading to severe consequences of COVID-19 disease [18–20]. Thus, proper infection prevention and control (IPC) measures form a critical part of HF readiness for providing high-quality delivery services.

Nepal faced an earthquake on a 7.8 Richter scale in 2015. In 2017, Nepal transitioned to a federal republic with the adoption of a new constitution in 2015 that divided the country into one federal, seven provincial, and 753 local governments. The constitution mandated that local governments provide basic healthcare services, including normal low-risk delivery services, free of cost to their people [21]. In this context, several policies seek to contribute to a reduction in the MMR and include the National Health Policy (2014), the Nepal Health Sector Strategic Plan (NHSSP) (2023–2030), and the Nepal Safe Motherhood and Newborn Health Road Map (2030). Additionally, the implementation of the National Health Care Quality Assurance Framework (2022) will guide both public and private sectors in the provision of quality maternal and newborn health services [22, 23]. Finally, there is a nationwide implementation of the maternity incentive scheme called “*Aama program*”, which ensures free delivery services and financial support through the provision of a transportation allowance to women who complete four scheduled antenatal visits and give birth at HFs.

While the increasing availability and use of HF-based delivery services represent important improvements, it is disconcerting that a large percentage of maternal deaths take place at HFs and that NMR are stagnating. This indicates that there may be a problem with the QoC of delivery services. This study aims to understand the supply-side issues among the HFs offering normal low-risk delivery services in Nepal. The primary objective is to assess changes in the HFs readiness to provide high quality in low risk deliveries and BEmONC services between 2015 and 2021. Secondary objectives are: the implementation of appropriate IPC in HFs providing delivery services, and to examine differences between public and private HFs regarding readiness and BEmONC service functionality. This study will have an impact on the health system by improving the HFs readiness on high quality delivery services by identifying areas lacking progress since 2015.

## Methods

### Data source

Data were drawn from the 2015 and 2021 Nepal Health Facility Surveys (NHFSs), with data being publicly available from https://dhsprogram.com/Data/. Both are comparable, nationally representative and cross-sectional surveys comprising the following components: inventory assessment; service provider interviews; observations of client-provider sessions for selected services; and exit interviews with patients or those taking care of patients for the selected services upon being discharged from a HF or leaving the service site. This study used data from the inventory assessment and delivery service provider interviews.

### Study population

This analysis comprised all formal sector HFs of Nepal that reported providing normal low-risk delivery services when data for the 2015 and 2021 NHFSs were collected. The NHFSs obtained their sampling frame from the Ministry of Health and Population of Nepal, which included 4,719 HFs in 2015 and 5,681 HFs in 2021 [12]. The increase in the number of HFs in 2021 was primarily due to the establishment of community health units by the local governments after the country’s health system was federalized in 2017. The NHFSs in 2015 and 2021 surveyed 940 and 1,564 HFs out of which 48.6% (457) and 51.4% (808) HFs respectively provided normal low-risk delivery services. The sample size of the NHFSs in 2015 and 2021 are comparable and allow for representative estimates nationally and by managing authority. The larger sample size in the NHFS 2021 accounts for the seven provinces of a federal Nepal, in contrast to the five administrative regions in 2015.

### Sampling

The HFs included in this analysis comprise all public hospitals offering basic healthcare services and all primary health care centers, and a sample of other basic healthcare centres (health/sub-health posts, community health units, and urban health centers). Similarly, for private HFs, this analysis in 2015 comprises sample of private hospitals with 15 or more in-patient beds, and complete enumeration of all private hospitals with 100 in-patient beds. In 2021, the analysis includes a sample of private hospitals with at least one in-patient bed, but complete enumeration of all private hospitals in the provinces which have fewer private hospitals.

### Analytical framework

The WHO Service Availability and Readiness Assessment (SARA) framework is used which is composed of three domains: i) staff and guidelines; ii) equipment; and iii) medicines and commodities [16]. To make the analysis of HF readiness for normal low-risk delivery services more granular and contextualised for Nepal, this study sub-divided the domain “staff and guidelines” into two distinct domains, i.e. “trained provider” and “guidelines”. The availability of guidelines merits to be assessed separately due to continuous changes in the evidence base informing health care delivery and related revisions of guidelines. Similarly, the domain “medicines and commodities” was sub-divided into a domain on “essential medicines for mothers”, and a domain on “essential medicines for newborns” to ensure adequate attention to newborn care. Each of these five domains received equal weight. For the BEmONC service functionality analysis, seven signal functions prediscribed by the SARA framework were used [16]. As the SARA framework does not contain a separate measure for IPC, this study applied selected domains of general HF readiness – i.e., i) trained provider, ii) guidelines, and iii) equipment and supplies – and used data on universal precaution measures and IPC available in the surveys. Each of the three domains received equal weight. This study focused on supply-side issues at the level of HFs and the domains recommended by WHO’s SARA manual. In contrast, further critical determinants of HF readiness according to the WHO health system building blocks framework, notably governance, information system and financing, where not explicitly addressed, as these primarily exert their influence at local or national government level, rather than at HF level.

### Study variables

For each HF, three summary indices were calculated and used as the main outcome variables in subsequent statistical analyses: (a) HF readiness index for normal low-risk delivery services; (b) BEmONC services functionality; and (c) IPC index.

*Original variables:* Tables 1, 2 and 3 show the original indicators and signal functions available in the NHFS 2015 and 2021 dataset for each SARA domain.

**Table 1:** Operational definition of HF readiness index for normal low-risk delivery services

*Domain/Index*	*Indicators analyzed from the NHFS 2015 and 2021 data set*	*Calculation*
Domain 1: Trained provider	*1 indicator –* At least one trained provider, regardless of the duration of training, is available to provide essential childbirth care regardless of the timing and duration of the training	Domain score = Indicator (0% = no provider, 100% = at least one provider)
Domain 2: Guidelines	*1 indicator –* Observed availability of at least one guideline on essential childbirth care, checklists and/or job aids for essential childbirth care	Domain score = Indicator (0% = no guideline, 100% = at least one guideline)
Domain 3: Equipment and supply	*13 indicators –* Observed availability and reported functionality of: i) emergency transport—this included ambulance or another vehicle for emergency transport, ii) delivery pack: a sterile delivery pack or all of the following 5 items: cord clamp, episiotomy scissors, scissors or blade, suture material with a needle, and needle holder, iii) examination light, iv) suction apparatus (mucus extractor), v) neonatal bag and mask, vi) delivery bed, vii) a blank partograph, viii) infant weighing scale, and ix) blood pressure (BP) set, x) latex gloves, xi) sterilization equipment, xii) manual vacuum extractor, and xiii) vacuum aspiration kit	Domain score = Percentage of functioning items available (Range: From 0% = no items to 100% = (at least 1 functioning unit of) all 13 items, e.g. 9/13 = 69.2% in the case of 9 functioning items available)
Domain 4: Essential medicines for mothers	*5 indicators –* Observed availability of at least one valid unit of: i) injectable uterotonic, ii) injectable antibiotic, iii) injectable magnesium sulfate, iv) skin disinfectant, and v) fluid with an infusion set	Domain score = Percentage of essential medicines for mothers available (Range: From 0% = no medicines to 100% = (at least 1 valid unit of) all 5 medicines, e.g. 4/5 = 80.0% in the case of 4 essential medicines for mothers available)
Domain 5: Essential medicines for newborns	*5 indicators –* Observed availability of at least one valid unit of: i) chlorhexidine gel, ii) tetracycline eye ointment, iii) injection gentamycin, iv) amoxicillin syrup, and v) ceftriaxone powder for injection	Domain score = Percentage of essential medicines for newborns available (Range: From 0% = no medicines to 100% = (at least 1 valid unit of) all 5 medicines, e.g. 2/5 = 40.0% in the case of 2 essential medicines for newborns available)
HF readiness index for normal low-risk delivery services	Readiness for normal low-risk delivery service is measured across 5 domains: i) trained provider, ii) guidelines, iii) equipment and supplies, iv) essential medicines for mothers, and v) essential medicines for newborns	HF readiness Index = Mean score of the five domain scores. (Range: From 0% = no readiness to 100% = complete readiness, e.g. (0% + 0% + 69.2% + 80.0% + 40.0%) / 5 = 37.8%)

**Table 2:** Operational definition of BEmONC service functionality

*Index*	*Signal functions analyzed the NHFS 2015 and 2021 data set*	*Calculation*
BEmONC service functionality	Reported performance of the following seven signal functions at least once during the three months before the survey—parenteral administration of: i) antibiotics, ii) oxytocin, and iii) anticonvulsants, iv) assisted vaginal delivery, v) manual removal of placenta, vi) removal of retained products of conception, and vii) neonatal resuscitation	BEmONC service functionality = Percentage of signal functions performed (Range: From 0% = none performed to 100% = all performed, e.g., 6/7 = 85.7% in the case of 6 signal functions performed)

**Table 3:** Operational definition of IPC index

*Domain/Index*	*Indicators analyzed the NHFS 2015 and 2021 data set*	*Calculation*
Domain 1: Trained provider	*1 indicator* – At least one provider available in the HF who is reported to be trained on IPC or waste management regardless of the timing and duration of the training	Domain score = Indicator (0% = no trained provider, 100% = at least one trained provider)
Domain 2: Guidelines	*1 indicator* – Observed availability of at least one guideline on health care waste management or infection control and prevention	Domain score = Indicator (0% = no guideline, 100% = at least one guideline)
Domain 3: Equipment and supplies	*10 indicators* – Observed availability and reported functionality of: i) soap and running water or alcohol-based hand rub, ii) needle destroyer, iii) waste receptacle, iv) disinfectant, v) gown or apron, vi) surgical mask, vii) latex gloves, viii) syringe, ix) eye protection, and x) sterilization equipment	Domain score = Percentage of functioning items available (Range: From 0% = no equipment and supplies to 100% = complete equipment and supplies, e.g. 6/10 = 60.0% in the case of 6 functioning items available)
IPC index	Readiness for IPC is measured across 3 domains: i) trained provider, ii) guidelines, and iii) equipment and supplies	IPC index = Mean score of the three domains (Range: From 0% = no IPC to 100% = complete IPC, e.g. (0% + 0% + 60.0%) / 3 = 20%)

*Calculation of domain scores and indices:* For each SARA domain, a domain score was calculated (see Tables 1, 2 and 3, column ‘Calculation’), ranging from 0 to 100%. At the end of each table, a summary index is described, calculated as the mean of the related domain scores, and ranging from 0 to 100%.

### Statistical analysis

Data analysis was conducted using IBM SPSS Statistics 25. Sample weights were applied to ensure the actual representativeness of findings at the national level and according to the managing authority. Initially, the weighted mean of the three outcome variables with their domains and sub-components, overall and stratified by the managing authority, was calculated for 2015 and 2021 respectively. Subsequently, a weighted t-test for independent samples was carried out to test for a statistically significant change over time in each of the outcome variables. The weighted mean difference of the change observed between 2015 and 2021 and its 95% confidence interval (CI) were calculated. A level of significance of 0.05 was assumed for all analyses.

### Ethical approval

The 2015 and 2021 NHFSs obtained ethical approval from the Nepal Health Research Council, while this study was approved both by the Ludwig-Maximilians-Universität (LMU Munich) Ethics Commission, Munich, Germany and by the Nepal Health Research Council in June 2021.

## Results

### Characteristics of HFs providing normal low-risk delivery services in Nepal

As shown in Table 4, this study analyzed 457 and 804 HFs providing normal low-risk deliveries in 2015 and 2021 respectively. In both years, more than eight out of ten HFs providing normal low-risk delivery services were public basic healthcare centres;

**Table 4:** (Weighted) distribution of health facilities providing normal low-risk delivery services in Nepal in 2015 and 2021 by health facility type and managing authority

**Background characteristics**	2015		2021	
	**Percent**	**Number**	**Percent**	**Number**
**Facility type**				
Public hospitals	4.4	20	5.2	42
Public basic healthcare centers	85.8	392	87.2	701
Private hospitals	9.8	45	7.6	61
**Managing authority**				
Public	90.2	412	92.4	743
Private	9.8	45	7.6	61
**Total health facilities**	**100.0**	**457**	**100.0**	**804**

### HF readiness for normal low-risk delivery services

**Fig. 2:**
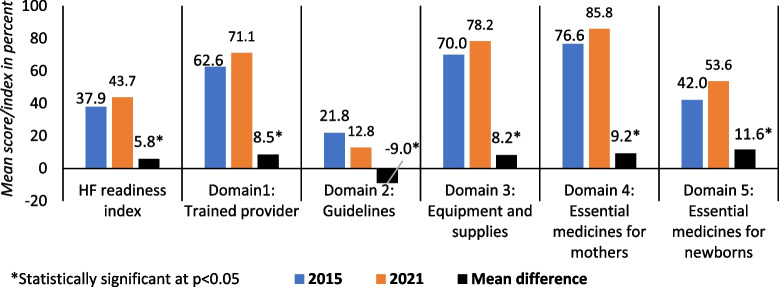
Health facility (HFs) readiness index for normal low-risk delivery services and domain-wise scores in Nepal in 2015 and 2021

As shown in Fig. 2, nationally, the average HF readiness index for normal low-risk delivery services was below 50% in both years despite a statistically significant increase from 37.9% in 2015 to 43.7% in 2021. There was, however, considerable variation between domain scores. Between 2015 and 2021, the greatest improvement was observed for essential medicines for newborns (statistically significant increase from 42.0% to 53.6%), followed by essential medicines for mothers (from 76.6% to 85.8%), equipment and supplies (from 70.0% to 78.2%), and trained providers (from 62.2% to 71.1%). Only one score showed a statistically significant decrease, i.e. the use of guidelines for essential delivery care decreased from 21.8% to 12.8%. Details are presented in Supplemental Table a.

Table 5 shows inter- and intra-domain variability of HF readiness. Availability of all 13 basic equipments and supplies for normal low-risk delivery services was excessively low and insignificantly increased from 6.5% in 2015 to 8.8% in 2021. Of the 13 items, the availability of nine significantly increased: emergency transport, examination light, delivery pack, neonatal bag and mask, delivery bed, partograph, blood pressure set, latex gloves, and infant weighing scale with levels in 2021 between 82.0% and 98.7%. The availability of the four remaining items – manual vacuum extractor, vacuum aspiration kit, suction apparatus and sterilization equipment – increased but not in a statistically significant manner.

**Table 5:** Availability of equipment and supplies, essential medicines for mothers and essential medicines for newborns among health facilities providing normal low-risk delivery services in Nepal in 2015 and 2021

	Mean in % (Percentage)		Mean difference in %	95% CI of mean difference in %	*P*-value
	**2015**	**2021**			
**Readiness domain: Equipment and supplies**					
i. Emergency transport	62.6	82.0	19.4	[14.2; 24.5]	< 0.0001*
ii. Examination light	60.7	93.8	33.1	[28.3; 37.9]	< 0.0001*
iii. Delivery pack	92.9	97.9	5.0	[2.3; 7.5]	< 0.0001*
iv. Suction apparatus	62.0	65.7	3.7	[-1.8; 9.3]	0.1890
v. Neonatal bag and mask	82.8	91.6	8.8	[4.9; 12.8]	< 0.0001*
vi. Delivery bed	96.3	98.7	2.4	[0.5; 4.3]	0.0140*
vii. Partograph	80.0	90.4	10.4	[6.2; 14.6]	< 0.0001*
viii. Blood pressure apparatus	84.1	95.3	11.2	[7.5; 14.8]	< 0.0001*
ix. Latex gloves	92.5	97.5	5.0	[2.3; 7.6]	< 0.0001*
x. Sterilization equipment	65.0	66.1	1.2	[-4.3; 6.7]	0.6690
xi. Infant weighing scale	89.9	94.1	4.2	[1.1; 7.5]	0.0090*
xii. Manual vacuum extractor	20.7	23.2	2.5	[-2.2; 7.2]	0.3030
xiii. Vacuum aspiration kit	19.2	20.9	1.7	[-2.8; 6.3]	0.4540
All 13 pieces of equipment	6.5	8.8	2.3	[-0.7; 5.3]	0.1320
**Readiness domain: Essential medicines for mothers**					
i. Injectable uterotonic	88.2	97.0	8.8	[5.6; 12.0]	< 0.0001*
ii. Injectable antibiotic	40.9	66.1	25.2	[19.6; 30.8]	< 0.0001*
iii. Injectable magnesium sulfate	72.2	70.7	-1.5	[-6.7; 3.7]	0.5700
iv. Skin antiseptic	91.4	98.1	6.7	[4.0; 9.5]	< 0.0001*
v. Intravenous solution with an infusion set	90.3	97.2	6.9	[3.9; 9.8]	< 0.0001*
All five essential medicines and commodities	29.7	49.8	20.1	[14.7; 25.6]	< 0.0001*
**Readiness domain: Essential medicines for newborns**					
i. 4% Chlorhexidine	58.0	80.2	22.2	[16.9; 27.5]	< 0.0001*
ii. Tetracycline eye ointment	39.5	7.8	-31.7	[-36.6; -26.8]	< 0.0001*
iii. Injection gentamycin	74.8	79.8	5.0	[0.2; 9.90]	0.0420*
iv. Amoxycillin syrup	25.7	62.2	36.5	[31.3; 41.80]	< 0.0001*
v. Ceftriaxone powder for injection	12.0	38.1	26.1	[21.6; 30.6]	< 0.0001*
All 5 essential medicines and commodities	0.7	2.2	1.50	[0.3; 2.8]	0.0180*
**Total health facilities**	**457**	**804**	**-**		**-**

^*^Statistically significant at *p* < 0.05

Nationally, one in two HFs (49.8%) had in stock all five essential medicines and commodities for mothers in 2021 which represents a significant increase from 29.7% in 2015. The availability of injectable uterotonics, skin antiseptics, and intravenous solutions with an infusion set was high, being in stock at nearly nine out of ten HFs in 2015, which improved significantly to nearly all HFs in 2021. Injectable antibiotics were available at two-thirds (66.1%) of facilities in 2021, representing a significant increase from 40.9% in 2015. Seven out of ten HFs stocked magnesium sulphate in both years. Compared to the availability of all five essential medicines and commodities for mothers, the availability of all five essential medicines for newborns in both surveys was extremely low and changed only slightly from 0.7% in 2015 to 2.2% in 2021. Stockout of tetracycline eye ointment was paramount, with only one in ten HFs having this stock in 2021. The availability of 4% chlorhexidine antiseptics, amoxicillin syrup, ceftriaxone injection, and injection gentamycin significantly increased between 2015 and 2021, ranging from 38.1% to 80.2% in 2021.

### BEmONC service functionality

Overall, at the national-level, functionality of BEmONC services was inadequate in HFs providing normal low-risk delivery services and did not show relevant improvements by 2021. As Table 6 shows, BEmONC service functionality was extremely low in both years, 4.2% in 2015 and 2.5% in 2021. Of the seven signal functions, the administration of parenteral oxytocin was high in both years and slightly increased to 88.2% by 2021, while all other signal functions decreased between 2015 and 2021. A significant decrease in functionality was found for assisted vaginal delivery, neonatal resuscitation, removal of retained products of conception, and manual removal of the placenta. The administration of anti-convulsants and assisted vaginal delivery were performed least frequently, below or at ten percent, among the seven signal functions in both years.

**Table 6:** BEmONC service functionality among health facilities providing normal low-risk delivery services in Nepal in 2015 and 2021

**Signal functions performed**	Mean in % (Percentage)		Mean difference in %	95% CI of mean difference in %	*p*-value
	**2015**	**2021**			
i. Administration of parenteral antibiotics	40.7	36.5	-4.2	[-9.9; 1.3]	0.1340
ii. Administration of parenteral oxytocin	85.8	88.2	2.4	[-1.5; 6.3]	0.2310
iii. Administration of parenteral anticonvulsants	10.0	8.9	-1.1	[-4.5; 2.3]	0.5290
iv. Assisted vaginal delivery	16.1	8.1	-8.0	[-11.8; -4.1]	0.0000*
v. Manual removal of placenta	42.8	36.7	-6.1	[-11.8; -0.5]	0.0340*
vi. Removal of retained products of conception	33.0	26.4	-6.6	[-11.9; -1.3]	0.0150*
vii. Neonatal resuscitation	36.8	29.6	-7.2	[-12.7; -1.8]	0.0090*
**All seven signal functions performed**	4.2	2.5	-1.7	[-3.9; 0.4]	0.1140
**Total health facilities**	**457**	**804**	**-**	**-**	**-**

^*^Statistically significant at *p* < 0.05

### Infection prevention and control

**Fig. 3:**
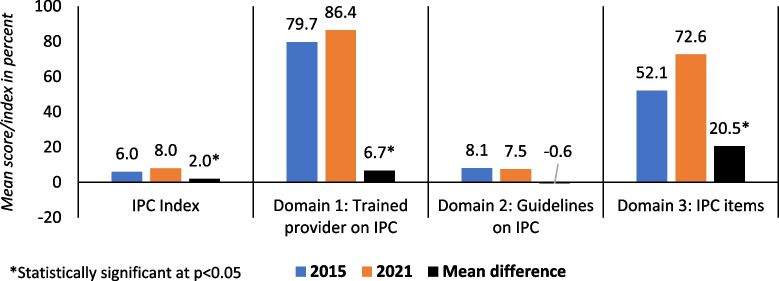
IPC index and domain-wise scores for health facilities providing normal low-risk delivery services for infection prevention and control in Nepal in 2015 and 2021

Figure 3 shows, on average, that the IPC index among HFs providing normal low-risk delivery services is very low in Nepal, despite a statistically significant increase from 6.0% in 2015 to 8.0% by 2021. Inter-domain variations were large. The availability of at least one provider trained regarding IPC had the highest score in both years and significantly increased from 79.7% in 2015 to 86.4% by 2021. The availability and functionality of IPC equipment and supplies had the largest increment in score (20.5% points), reaching 72.6% by 2021. The availability of guidelines on IPC was below ten percent in both years, which significantly lowered the overall IPC index. Details are presented in Supplemental Table b.

The availability of all ten items under IPC equipment and supplies increased significantly from 0.7% in 2015 to 6.3% by 2021 (mean difference: 5.5, CI: [3.7; 7.4], *p* < 0.0001), however, these levels are far too low to ensure appropriate IPC measures. The availability of all four items of personal protective equipment – latex gloves, surgical masks, eye protection, and gowns or aprons – significantly increased from a mere 5.9% in 2015 to 51.1% in 2021 (mean difference: 45.3, CI: [41.2; 49.3], *p* < 0.0001), however, this level is still considered inadequate, particularly for eye protection supplies.

### Key findings by managing authority

The HF readiness index for normal low-risk delivery services showed statistically significant increases from 37.6% to 43.6% among public HFs, and from 39.9% to 45.3% among private HFs between 2015 and 2021 respectively, thus showing small differences by managing authority. Differences regarding the changes in HF readiness domains between public and private HFs are reported in Supplemental Table a.

In 2021, the disparity in BEmONC service functionality of HFs providing normal low-risk delivery services by managing authority was large: public 1.8% and private 10.5%, which was a decline from 3.0% in public and from 15.7% in private HFs from 2015 respectively. The statistically significant changes in two signal functions among the public HFs are reported in Supplemental Table c.

The IPC index was found to be low for both managing authorities, with a slightly better score among private HFs (6.9% in 2015, 8.4% in 2021) than among public HFs (5.9% in 2015, 7.9% in 2021). The statistically significant changes in domain level scores between public and private HFs are reported in Supplemental Table b.

## Discussion

### Key findings and locating them in the literature

In the context of the constitutional right of the Nepali people to free basic healthcare services, the mandate for local governments to manage their basic healthcare services, the inclusion of normal-low risk delivery services into the basic healthcare package, and a strategic focus on improving the QoC, the present study analyzed nationally representative data from HFs providing low-risk delivery services with regard to their readiness, functionality of basic emergency obstetric and newborn care services, and infection prevention and control.

### Health facility readiness for normal low-risk delivery services- quality of care

The readiness of HFs in Nepal for normal, low-risk delivery services is suboptimal. While there have been improvements in the HF readiness index from 37.9% in 2015 to 43.7% in 2021, these changes are small and equate to a 0.9% annual improvement rate. This indicates that the majority of HFs in Nepal have low scores for HF readiness. A number of studies published using the NHFS 2015 data identifies inadequate number of service providers, irregular services, supply chain issues being key challenges for continuum and quality of delivery care across both public and private HFs [24, 25]. The low readiness of HFs to provide high-quality, normal, low-risk delivery services is not surprising because the QoC for other basic healthcare services is also sub-optimal. For instance, less than two percent of HFs meet minimum standards of care for antenatal, family planning, and sick child services [12, 26]. Similar results have been observed in other low- and middle-income countries (LMICs). For example, in Nigeria, between 2005 and 2009, there was no statistically significant progress in the availability of trained providers for labor and delivery care (from 50.8% to 57.1%). Indeed, the readiness of HFs to provide maternal health services was low in both years [27]. One major challenge for LMICs is the lack of commonly agreed upon QoC indicators, which hinders measuring progress nationally and makes it difficult to compare indicators across countries. The use of the Minimum Service Standard (MSS) tool in public HFs is a relatively new concept but its use has now standardized monitoring of HF readiness in Nepal. The reporting of MSS scores as part of the routine Health Management Information System database has helped local governments and HFs identify gaps in HF readiness, link them with service utilization data, and take corrective actions. This tool has led to positive changes and that policy implications are to strengthen this standardized monitoring.

In this study, wide variation across the five domains of HF readiness was found between the survey years. Four out of five domains, namely equipment and supplies, essential medicines for mothers, trained providers, and essential medicines for newborns, significantly improved in 2021 with large variations within the components. This could be due to the focus on improving QoC during the implementation of the Nepal Health Sector Strategy (NHSS) 2016–2022. In contrast, the fifth domain, the availability of guidelines on delivery care, performed the least and significantly decreased from 21.8% in 2015 to 12.8% in 2021. This is low compared to other LMICs such as Haiti and Tanzania where delivery care guidelines were available in 24.1% and 29.8% of the HFs in 2017 and 2019, respectively [26, 28]. The low and diminished availability of guidelines in 2021 in Nepal could be due to COVID-19 restrictions, resulting in a largely virtual mode of operations and halting the printing of guidelines or their dissemination to HFs. Nevertheless, during the COVID-19 pandemic, digital guidelines, particularly on COVID-19 topics, were provided to HFs. Program guidelines comprise evidence-informed recommendations intended to standardize and optimize care for patients, and their use during clinical decision-making is intended to improve healthcare outcomes [29, 30]. Even though both paper and digital guidelines were included in this analysis, the low results in 2021 suggest little or no effort made to improve guideline availability since 2015. Anecdotal evidence indicates improper storage of digital guidelines in electronic devices, that cannot be accessed by service providers when needed. Improving the skills of providers in accessing these guidelines on computers and mobile devices can support increased use of the digital guidelines. The COVID-19 pandemic disrupted regular trainings [31, 32], but Nepal was able to significantly increase the availability of trained delivery service providers by 2021. Reaching the SDG target of attaining 90% skilled birth attendance was a focus of NHSS (2016-2022), and several training packages were available to train the delivery care service providers on enhancing their knowledge and skills, which were continued virtually during COVID-19. However, gaps still exist, with nearly one in three HFs lacking a provider trained in delivery care. Human resource availability and their skills remain a significant health system challenge that is not unique to Nepal but relevant for many other LMICs [33].

Supply chain issues have emerged, particularly with essential medicines for newborns and basic equipment and supplies. The frequent lack of equipment could be a reason for the low rates of assisted deliveries in HFs. Additionally, the low availability of sterilization equipment increases the risk of nosocomial infections for both the mother and the newborn, and the low availability of tetracycline ointment for the treatment of newborn eye infections illustrates poor readiness for newborn care. This is alarming since the NMR has not been reduced below 21 deaths per 1,000 live births since 2016 [6]. Although the stock of essential medicines for mothers was better than for newborns in both managing authorities, there is still a significant lack of supply, mainly for injectable magnesium sulphate and injectable antibiotics. This is alarming because in 2021, 12.0% and 11.2% of maternal deaths during the post-partum period were due to hypertensive disorders and infections respectively; and 10.2% of the maternal deaths during delivery had fits, seizures, and convulsions [2]. Data from 28 hospitals over the four-year period from 2015 to 2018 reports eclampsia to be the leading cause of maternal deaths (19.0%). The availability of magnesium sulphate in LMICs is consistently low. A study published in 17 LMICs in 2018 revealed that magnesium sulphate was amongst the least available essential medicines and commodities for mothers (median: 63%, range: 10.0–97.0%) [34]. It is important to ensure a reliable supply of magnesium sulfate and antibiotics and enforce its correct use in training. The supply chain system of any country is affected by the service delivery structure. Prior to Nepal being declared a federal republic in 2017, medicines were procured by the central government system. Now, all three tiers of government procure medicines, but there is a lack of clarity on which level of government should be responsible for which task. As a result, there is often over or understocking of medicines at HFs. The overall capacity of local governments to procure medicines through systematic data-driven forecasting and following public procurement guidelines is limited. While both push and pull supply-chain systems are in place, establishing a strong supply chain system remains an ongoing challenge. Data-driven procurement, efficient transportation management, and improving governance at all levels should be prioritized.

### Basic emergency obstetric and neonatal care (BEmONC) service functionality

Fifteen percent of pregnant women develop a potentially life-threatening complication that requires skilled medical care, and some may even require major obstetrical intervention for survival [35]. According to verbal autopsy reports on maternal deaths conducted in Nepal in 2021, the majority of maternal deaths occur during the postpartum period due to obstetric hemorrhage and take place at HFs [2]. This underscores the importance of functional BEmONC services. Sadly, in 2021, only 2.5% of HFs had functional BEmONC services, indicating a decline from 4.5% reported in 2015, which may be attributed to health system challenges arising from the COVID-19 pandemic such as HF closures or disfunctional supply-chain systems. In 2022, eight out of ten deliveries at HFs were attended by skilled birth attendants at HFs [1]. This means that the majority of HFs can provide assisted vaginal delivery services to pregnant women in need. However, in 2021, only 16.2% of HFs offering normal low-risk deliveries reported having assisted vaginal delivery services available. Private HFs consistently had higher BEmONC service functionality in both surveys than public HFs; this could be because all private HFs analyzed in this study were hospitals, unlike only about 5% of the public HFs analyzed. Studies on BEmONC in Africa and Pakistan have identified a shortage of trained providers, high absenteeism rates, poor ambulance services, and supply chain issues as barriers to quality BEmONC services [28, 36]. These barriers are also relevant in the Nepalese context. Other obstacles to quality BEmONC in LMICs include poor provider remuneration and demoralization, high turnover, increased workload and burnout rates, poor coordination, inefficient referral mechanisms, inadequate allocation of limited resources, lack of training and monitoring, and inequality in the distribution of BEmONC sites [37]. In Nepal, under the *Aama Program*, HFs receive unit costs for attending normal-low-risk, complicated, and cesarean deliveries, a small amount of which is provided to the birth attending team, which can motivate the providers [38]. However, this study did not analyze the barriers and the motivation factors in depth.

With improved access to HF-based delivery services, eight out of ten women giving birth in HFs [6] and over half of the maternal deaths occurring in HFs, the low functionality of the BEmONC service and lack of progress are alarming in fulfilling the SDG commitment of reducing maternal deaths. The low volume of deliveries in basic healthcare centers, due to improper location and inadequate HF readiness, results in overcrowding in hospitals [0 24]. Considerable home deliveries, lack of awareness of the *Aama Program*, and the impact of the COVID-19 pandemic for 2021 could explain the low BEmONC service functionality in Nepal. Furthermore, these findings reinforce the thrust of the Nepal Safe Motherhood and Newborn Health Road Map (2030) and NHSSP 2023–2030 and suggest re-thinking the strategic locations of the HFs providing BEmONC services, and creating demand for those HFs, as well as improving services, including staffing in hospitals.

### Infection prevention and control

Universal precautions for IPC are important not only in the face of the COVID-19 pandemic and other disease outbreaks but also in routine healthcare provision. Birth itself and vaginal examinations make pregnant women more susceptible to infections. Despite significant improvements in the IPC index between 2015 and 2021 found in this study, the 2021 IPC levels of 7.9% for public HFs and 8.4% for private HFs are excessively low. Although the availability of trained providers on IPC and personal protective equipment significantly improved in 2021 due to COVID-19 response efforts, the availability of IPC guidelines is also low, and the overall IPC status is inadequate. The low readiness for IPC found in this study is comparable to HFs in Bangladesh, where only 16.4% of the standard precaution elements were available for delivery and newborn care services in 2017 [29], and another analysis of 17 LMICs where the median national availability of sterilization equipment was 51.0% in 2018 [34]. Items such as waste receptacles and needle destroyers were often not available in HFs, indicating a need to strengthen supply chain systems and put into place quality improvement teams [13, 39]. In the new federal system in Nepal, resources can be leveraged from local governments for local purchase of these easily available items.

### Strengths and limitations of the study

This study has both strengths and limitations. It is the first study to draw on data from two nationally representative NHFSs implemented under the global Demographic and Health Survey (DHS) Program to measure changes in HF readiness for normal low-risk deliveries. This occurred over a time period when Nepal was hit by a 7.8 Richter Scale earthquake and the recent COVID-19 pandemic and amid the country's transition to a federal government system. Methodologically, the NHFS derived a sampling frame from Nepal's national health system that included both public and private HFs. The survey instruments were validated, and verbal responses were confirmed by observations. Data were collected in tablets by thoroughly trained medical, nursing, or public health personnel. Data quality was checked daily by the local research firm and the DHS Program, and the analysis was guided by publicly available code books, weighing techniques, and tutorials at https://dhsprogram.com/Data/. Although data collection for the 2015 and 2021 NHFSs was impacted by the earthquake and the COVID-19 pandemic, respectively, standard operating procedures were fully followed, and there were no major quality concerns except that data collection was delayed and took longer than planned. However, there are some limitations. These include, for example, the small sample size for private HFs since only private hospitals were surveyed, and the fact that stock data were impacted by the timing of receipt of the stocks at HFs, and that the 2015 earthquake and the COVID-19 pandemic made supply chains vulnerable and irregular. General amenities like waiting areas, sanitation, and drinking water facilities were not analyzed, and newborn care was not comprehensively covered in the analysis. This study focused only on the variables recommended by WHO’s SARA manual and did not analyze other health system factors.

### Implications for policy, practice and research

The policy environment for improving QoC is progressing in Nepal. However, several challenges exists that need to be addressed. For instance, the establishment of an accrediting body for quality assurance in the public and private health sector, as envisioned in the NHSSP (2023–2030), has not been fully effective yet; local governments have limited human resource capcity to provide basic healthcare services; monitoring mechanisms for basic healthcare services remain unclear; and the lack of accountability mechanisms is hindering progress which impacts the HF readiness to provide delivery services. The standards for normal low-risk deliveries are equal for all HFs regardless of their size and managing authority [16, 23, 40, 41]. While the private sector in Nepal is expanding, it represents the first point of contact for women seeking delivery services in several parts of the country [1] but monitoring QoC in the private sector is challenging due to unclear regulations. Comparatively, private HFs have slightly better readiness than public HFs, with better availability of essential medicines and equipment. However, they lack adequately trained providers and delivery care guidelines. While they are more ready to provide BEmONC services, and generally more resourceful compared with public HFs, they need a better operating environment and a motivation to implement the *Aama Program*, which can create a win–win situation for both the public and the private sectors, ensuring high QoC for pregnant women.

The earthquake in 2015 claimed the lives of over 9,000 people, while the COVID-19 pandemic resulted in a death toll of over 12,000 people [42] and continues to impact Nepal's health system. Although Nepal's transition from a unitary to a federal country allowed for local planning and management of basic healthcare services, unclear demarcation of authority between jurisdictions in the three tiers of government led to slow decision-making and adversely affected healthcare service delivery [43]. This study indicates that, following the shift to a federal system, increased emphasis was placed on expanding the number of HFs, but not enough attention was paid to improving their readiness and the QoC. The challenges of inadequate and insufficiently skilled staff at HFs need to be addressed by local governments through robust planning, budgeting, implementation, and oversight of basic healthcare services. Similarly, clarifying the roles and responsibilities of the three tiers of government in the delivery of basic healthcare services and fostering proper coordination among them remain crucial to ensure HF readiness for high-quality services, including normal low-risk births, obstetric complication management, and improved maternal survival.

Data availability for QoC is improving in Nepal [12]. The implementation of previous health sector strategies has informed the government and other stakeholders to develop the NHSSP (2023–2030), the Nepal Safe Motherhood and Newborn Health Road Map 2030, and a nationally representative, comprehensive NHFS to monitor changes in QoC at five-year intervals. The NHFS 2021 is the key data sources to track HF readiness indicators of the NHSSP (2023–2030), its continuity will provide opportunities for researchers and policymakers to analyze trends and rates of changes in HF readiness for delivery and other basic healthcare services. However, Nepal also needs a functional information system that can routinely track QoC indicators in both public and private sectors. While the Health Management Information System and Logistic Management Information System monitor service utilization rates, medicine stock, and Minimum Service Standard scores at HFs, they are limited in monitoring other important QoC indicators.

Improving QoC needs to remain a top priority in the health system in Nepal, with clear roles, evidence-based strategies, and interventions designed for all providers, including the three tiers of government and the private sector. The gaps in HF readiness identified in this study, as well as those in NHFS 2021, the Minimum Service Standard tool implementation in public HFs, and other studies, need to be collated to generate knowledge and translate them into budgeted plans for the relevant governments. These actions should ensure effective supply chain management, training and regular availability of service providers, availability of program guidelines, and functioning quality improvement systems. The local governments and HFs need to leverage internal and external resources and monitor the effective implementation of basic healthcare services, which are key to increasing HF births, managing obstetric complications, preventing women from nosocomial infections, and ultimately reducing maternal deaths. The results of this study can complement the baseline and review of the result framework for the NHSSP (2023–2030), with suggested QoC indicators, monitoring approaches, and support for tracking progress over time.

## Conclusions

The slow progress and sub-optimal readiness for normal, low-risk deliveries and IPC, along with declining and low BEmONC service functionality in 2021, is reflective of poor QoC and provides some proximate explanation for the moderately high maternal mortality and the stagnation of NMR in Nepal. However, these findings need to be interpreted within the context of the effects of the 2015 earthquake, the COVID-19 pandemic, and the new federal government system in Nepal. Despite QoC being a strategic focus of the NHSS (2016–2022) and HF readiness being a prerequisite for ensuring the process of care provision and client satisfaction, there are gaps in the current supply chain system, human resource capacity, quality improvement approaches adopted, and the operating environment for the private sector. To fulfil the constitutional mandate of managing basic healthcare services and providing high-quality delivery services to pregnant women, improving the capacity of local governments is critical, ultimately contributing to meeting SDG 3.1 target of reducing maternal deaths.

### Supplementary Information

**Additional file 1: Supplemental Table a.** Health facility readiness index and domain-wise scores of HFs to provide normal low-risk delivery services in Nepal in 2015 and 2021 in total and by managing authority. **Supplemental Table b.** Infection prevention and control index and domain-wise score for of health facilities providing normal low-risk delivery services in Nepal in 2015 and 2021, in total and by managing authority. **Supplemental Table c.** BEmONC service functionality among health facilities providing normal low-risk delivery services in Nepal in 2015 and 2021, by managing authority.

## Data Availability

The data used in the study is publicly available at the DHS Program website: https://dhsprogram.com/Data/.

## Abbreviations

SDG: Sustainable Development Goal
HF: Health facility
BEmONC: Basic emergency obstetric and neonatal care
EmONC: Emergency obstetric and neonatal care
UN: United Nations
NMMS: Nepal Maternal Mortality Study
NMR: Neonatal Mortality Rate
DHS: Demographic and Health Survey
WHO: World Health Organization
QoC: Quality of Care
COVID: Corona virus disease
IPC: Infection prevention and control
NHSS: Nepal Health Sector Strategy
NHSSP: Nepal Health Sector Strategic Plan
NHFS: Nepal Health Facility Survey
SARA: Service Aaialbility and Readiness Assessment
SPSS: Statistical Package for the Social Sciences
LLMU: Ludwig-Maximilians-Universität
CI: Confidence interval
LMIC: Low and middle income countries
MSS: Minimum Service Standards

## References

[1] Ministry of Health (MOH) Nepal; New ERA, Nepal; ICF. Nepal Demographic and Health Survey 2016. Kathmandu: Ministry of Health; 2017.

[2] Ministry of Health and Population (MOHP) and National Statistics Office (NSO). National population and housing census 2021. A report on maternal mortality. Government of Nepal. 2022. Available from https://mohp.gov.np/uploads/Resources/Final%20Report-26%20March-%202023-UPDATED.pdf. https://www.dhsprogram.com/pubs/pdf/fr336/fr336.pdf.

[3] Malla DS, Giri K, Karki C, Chaudhary P (2011). Achieving Millennium Development Goals 4 and 5 in Nepal. BJOG..

[4] National Planning Commission (NPC). Nepal’s Sustainable Development Goals Status and Roadmap: 2016–2030. Published by Government of Nepal, National Planning Commission, Singha Durbar, Kathmandu Nepal. 2017. Available from http://sdg.npc.gov.np/media/resources/items/0/bSustainable_Development_Goals_Status_and_Roadmap__2016-2030_46E6XzP.pdf.

[5] Bhutta ZA, Das JK, Bahl R (2014). Can available interventions end preventable deaths in mothers, newborn babies, and stillbirths, and at what cost?. Lancet.

[6] Ministry of Health and Population (MOHP) Nepal, New ERA, Nepal; ICF. Nepal Demographic and Health Survey (NDHS) 2022. Ministry of Health and Population, Kathmandu, Nepal. 2022. Available from The DHS Program. Nepal: DHS; 2022. Final Report (English).

[7] ICF. The DHS Program STATcompiler. Funded by USAID. 2012. Available from http://www.statcompiler.com. February 28, 2023.

[8] World Health Organization (WHO). Care in normal birth: a practical guide. report of a technical working group. Reproductive health and research. Geneva: WHO; 1996. https://cdn1.sph.harvard.edu/wp-content/uploads/sites/2413/2014/08/WHO_FRH_MSM_96.24.pdf.

[9] Department of Health Services (DOHS). Annual Report 2020/2021. Government of Nepal. Kathmandu: Ministry of Health and Population. https://dohs.gov.np/annual-report-fy-2077-78-2019-20/.

[10] World Health Organization (WHO) (2006). Quality of Care: A Process for Making Strategic Choices in Health Systems.

[11] Kruk ME, Gage AD, Arsenault C, Jordan K, Leslie HH, Roder-DeWan S, Adeyi O, Barker P, Daelmans B, Doubova SV, English M, García-Elorrio E, Guanais F, Gureje O, Hirschhorn LR, Jiang L, Kelley E, Lemango ET, Liljestrand J, Malata A, Pate M (2018). High-quality health systems in the Sustainable Development Goals era: time for a revolution. Lancet Glob health.

[12] Ministry of Health and Population (MOHP), Nepal. New ERA, Nepal, ICF. Nepal Health Facility Survey 2021. 2022. Available from https://dhsprogram.com/pubs/pdf/SPA35/SPA35.pdf.

[13] World Health Organization (WHO). Standards for improving quality of maternal and newborn care in health facilities. Printed by the WHO Document Production Services, Geneva, Switzerland. 2016. Available from https://cdn.who.int/media/docs/default-source/mca-documents/qoc/quality-of-care/standards-for-improving-quality-of-maternal-and-newborn-care-in-health-facilities.pdf.

[14] World Health Organization (WHO). Quality of care for maternal and newborn health: a monitoring framework for network countries. February 2019. Department of Maternal, Newborn, Child and Adolescent Health (MCA), World Health Organization HQ Avenue Appia 20, 1211 Genève, Switzerland. Available from https://cdn.who.int/media/docs/default-source/mca-documents/qoc/qed-quality-of-care-for-maternal-and-newborn-health-a-monitoring-framework-for-network-countries.pdf?sfvrsn=19a9f7d0_1.

[15] Loporto. J Soc Change. 2020;12(1):40-70. 10.5590/JOSC.2020.12.1.05. https://scholarworks.waldenu.edu/cgi/viewcontent.cgi?article=1252&context=jsc.

[16] World Health Organization (WHO). 2015. Service Availability and Readiness Assessment (SARA): an annual monitoring system for service delivery, reference manual. Version 2.2. Revised July 2015. Avaialble from https://apps.who.int/iris/bitstream/handle/10665/149025/WHO_HIS_HSI_2014.5_eng.pdf?sequence=1&isAllowed=y

[17] Management Division. Assess impact of COVID-19 pandemic in selected health services with estimation of ‘excess maternal deaths’. Government of Nepal Ministry of Health and Population Department of Health Service Management Division Integrated Health Information Management Section Kathmandu, Nepal. 2021. https://dohs.gov.np/wp-content/uploads/2021/09/Impact-of-COVID-19-on-selected-health-services.pdf.

[18] Hongbo Q, Miaomiao C, Xin L, Xiyao L, Yuan S, Tianjiao L, Hua Z, Jun Z, Yangyu Z, Chao T, Philip NB (2020). Management of a delivery suite during the COVID-19 epidemic. Eur J Obstet Gynecol Reprod Biol.

[19] Aaron N, Michael LN, Andrew WM, Gary LG, Santiago JM, Ernesto M (2022). Nosocomial COVID-19 infection in women undergoing elective cesarean delivery: a prospective cohort study. Am J Obstet Gynecol MFM..

[20] Abbas M, Robalo NT, Martischang R, et al. Nosocomial transmission and outbreaks of coronavirus disease 2019: the need to protect both patients and healthcare workers. Antimicrob Resist Infect Control. 2021;10:7. 10.1186/s13756-020-00875-7. 10.1186/s13756-020-00875-7 PMC7787623 33407833

[21] Curative Division (CD). Basic Healthcare Service. Ministy of Health and Population. 2018. Available from https://publichealthupdate.com/basic-health-service-package-2075-dohs-mohp-nepal/.

[22] Ministry of Health and Population (MOHP). Nepal Health Sector Strategic Plan 2023–2030. Ramshahpath, Kathmandu: Government of Nepal, Ministry of Health and Population; 2023.

[23] Family Welfare Division (FWD) (2019). Nepal Safe Motherhood and Newborn Health Roadmap 2019.

[24] Acharya K, Subedi RK, Dahal S (2021). Basic emergency obstetric and newborn care service availability and readiness in Nepal: analysis of the 2015 Nepal Health Facility Survey. PLoS One.

[25] Khatri RB, Assefa Y, Durham J (2022). Assessment of health system readiness for routine maternal and newborn health services in Nepal: analysis of a nationally representative health facility survey, 2015. PLOS Glob Public Health..

[26] Wenjuan W, Michelle W, Clara RB (2017). Limited service availability, readiness, and use of facility-based delivery care in haiti: a study linking health facility data and population Data. Glob Health Sci Pract.

[27] Gage AJ, Ilombu O, Akinyemi AI (2016). Service readiness, health facility management practices, and delivery care utilization in five states of Nigeria: a cross-sectional analysis. BMC Pregnancy Childbirth.

[28] Bintabara D, Ernest A, Mpondo B (2019). Health facility service availability and readiness to provide basic emergency obstetric and newborn care in a low-resource setting: evidence from a Tanzania national survey. BMJ Open.

[29] Pantoja T, Soto M (2014). Clinical practice guidelines development and implementation: an introduction. Rev Med Chil.

[30] O’Neill K, Takane M, Sheffel A, Abou-Zahr C, Boerma T (2013). Monitoring service delivery for universal health coverage: the service availability and readiness assessment. Bull World Health Organ.

[31] Gyanwali P, Bista NR, Khadka M, Vaidya A, Mahato NK, Karn MK, Pant S, Ghimire N, Pokhrel A, Dhimal M (2021). Assessment of preparedness of government of Nepal in COVID designated hospitals and clinics for pandemic response. J Nepal Health Res Counc.

[32] Giri S, Panth A (2022). Knowledge and Perceptions of Corona Virus Disease (COVID 19) among Nurses Working at Teaching Hospital, Banke, Nepal.

[33] Bolan N, Cowgill KD, Walker K, Kak L, Shaver T, Moxon S, et al. Human Resources for Health-Related Challenges to Ensuring Quality Newborn Care in Low- and Middle-Income Countries: A Scoping Review. Glob Health Sci Pract. 2021;160–76. 10.9745/ghsp-d-20-00362. 10.9745/GHSP-D-20-00362 PMC8087437 33795367

[34] Kanyangarara M, Chou VB, Creanga AA, Walker N (2018). Linking household and health facility surveys to assess obstetric service availability, readiness and coverage: evidence from 17 low- and middle-income countries. J Glob Health.

[35] World Health Organization (WHO). Monitoring emergency obstetric care: a handbook. WHO Library Cataloguing-in-Publication. 2009. Available from https://apps.who.int/iris/bitstream/handle/10665/44121/9789241547734_eng.pdf?sequence=1&isAllowed=y

[36] Utz B, Zafar S, Arshad N, Kana T, Gopalakrishnan S; Nynke van den Broek. Status of emergency obstetric care in four districts of Punjab, Pakistan - results of a baseline assessment. J Pak Med Assoc. 2015;65(5):480-5. PMID: 26028380. 26028380

[37] Chi PC, Bulage P, Urdal H, Sundby J (2015). Barriers in the delivery of emergency obstetric and neonatal care in post-conflict Africa: qualitative case studies of Burundi and Northern Uganda. PLoS One.

[38] Mother and Newborn Program Guidelines 2021 (Aaman tatha Nawajat Sishu Surakshya Karyakram 2078 Nidershika) Nepali. Government of Nepal, Ministry of Health and Population, Department of Health Services, Family Welfare Division, Teku, Kathmandu, Nepal. https://digitallibrary.fwd.gov.np/list/13?categoryName=MATERNAL+%26+NEWBORN+HEALTH&subCategoryName=Safe+Motherhood.

[39] Md A, Md ZH, Mohammad RM, Md SI, Aninda R, Zubair A, Fahmida C, Sayera B, Nusrat H. Assessment of standard precaution related to infection prevention readiness of healthcare facilities in Bangladesh: Findings from a national cross-sectional survey PhD6,7. Antimicrob Steward Healthc Epidemiol. 2021;1:e52, 1–7. 10.1017/ash.2021.226. 10.1017/ash.2021.226 PMC9495545 36168506

[40] Dominic M, Katie G, Michelle KN, Kali PR, Ananta BS, Kovid S, Cathy G, May S. Results of a person-centered maternal health quality improvement intervention in Uttar Pradesh, India, Published: December 11, 2020. 10.1371/journal.pone.0242909. 10.1371/journal.pone.0242909 PMC7732121 33306689

[41] Family Welfare Division (FWD). National Medical Standard (NMS) Volume III. Ministry of Health and Population. 2019. Available from https://nhssp.org.np/Resources/SD/NMS%20for%20Maternal%20&%20New%20Born%20Care%20-%20PD%20R6%20-%20July%202020.pdf.

[42] Ministry of Health and Population (MOHP). Government of Nepal SitRep# 1052. 2022. Available from https://covid19.mohp.gov.np/covid/englishSituationReport/63aad308882e1_SitRep1052_COVID-19_27-12-2022_EN.pdf.

[43] Thapa R, Bam K, Tiwari P, Sinha TK, Dahal S. Implementing federalism in the health system of Nepal: opportunities and challenges. Int J Health Policy Manag. 2019;8(4):195–8. 10.15171/ijhpm.2018.121. 10.15171/ijhpm.2018.121 PMC6499910 31050964

