# Supplementary material for: Changes in health facility readiness for obstetric and neonatal care services in Nepal: an analysis of cross-sectional health facility survey data in 2015 and 2021
Source: BMC Pregnancy Childbirth. 2024 Jan 24;24:79. doi: 10.1186/s12884-023-06138-8 (PMC10807104; doi:10.1186/s12884-023-06138-8)
Supplement: Supplementary file 1 — Additional file 1: Supplemental Table a. Health facility readiness index and domain-wise scores of HFs to provide normal low-risk delivery services in Nepal in 2015 and 2021 in total and by managing authority. Supplemental Table b. Infection prevention and control index and domain-wise score for of health facilities providing normal low-risk delivery services in Nepal in 2015 and 2021, in total and by managing authority. Supplemental Table c. BEmONC service functionality among health facilities providing normal low-risk delivery services in Nepal in 2015 and 2021, by managing authority. [file 12884_2023_6138_MOESM1_ESM.docx]

**Supplemental Table a**

| Table: Health facility readiness index and domain-wise scores of HFs to provide normal low-risk delivery services in Nepal in 2015 and 2021 in total and by managing authority | | | | | |
| --- | --- | --- | --- | --- | --- |
| Readiness Index and domain scores | **Mean score/index in %** | | **Mean difference in %** | **95% CI of mean difference in %** | **p-value** |
|  | **2015** | **2021** |  |  |  |
| Health Facility Readiness Index | **37.9** | **43.7** | **5.8** | **[4.8; 6.8]** | **<0.0001*** |
| Public | **37.6** | **43.6** | **6.0** | **[4.9; 6.9]** | **<0.0001*** |
| Private | **39.9** | **45.3** | **5.4** | **[0.6; 10.2]** | **0.0280*** |
| Domain 1: Provider trained on delivery care | 62.6 | 71.1 | 8.5 | [3.0; 13.9] | 0.0020* |
| Public | 65.7 | 73.4 | 7.7 | [2.2; 13.3] | 0.0070* |
| Private | 34.9 | 43.2 | 8.3 | [-10.8; 27.4] | 0.3920 |
| Domain 2: Guidelines for essential delivery care | 21.8 | 12.8 | -9.0 | [-13.5; -4.6] | <0.0001* |
| Public | 24.1 | 13.2 | -10.9 | [-15.7; -6.1] | <0.0001* |
| Private | 0.5 | 7.4 | 6.9 | [-0.10-13.9] | 0.0530 |
| Domain 3: Equipment and supplies | 70.9 | 78.2 | 8.2 | [6.5; 10.1] | <0.0001* |
| Public | 68.7 | 77.6 | 8.9 | [7.1; 10.7] | <0.0001* |
| Private | 80.8 | 85.5 | 4.7 | [-3.4; 12.9] | 0.2470 |
| Domain 4: Essential medicines for mothers | 76.6 | 85.8 | 9.2 | [6.8; 11.6] | <0.0001* |
| Public | 76.8 | 86.1 | 9.3 | [6.9; 11.7] | <0.0001* |
| Private | 74.6 | 82.2 | 7.6 | [-4.6; 19.8] | 0.2190 |
| Domain 5: Essential medicines for newborns | 42.0 | 53.6 | 11.6 | [9.2; 14.0] | <0.0001* |
| Public | 41.8 | 53.2 | 11.4 | [9.0; 13.9] | <0.0001* |
| Private | 43.9 | 58.3 | 14.4 | [3.4; 25.3] | 0.0110* |
| Total health facilities | **457** | **804** |  | **-** | **-** |
| *Significant at p<0.05 | | | | | |

**Supplemental Table b**

| Table 9: Infection prevention and control index and domain-wise score for of health facilities providing normal low-risk delivery services in Nepal in 2015 and 2021, in total and by managing authority | | | | | |
| --- | --- | --- | --- | --- | --- |
| Readiness Index and domain scores | **Mean score/index in %** | | **Mean difference in %** | **95% CI of mean difference in %** | **p-value** |
|  | **2015** | **2021** |  |  |  |
| Infection prevention and control index | **6.0** | **8.0** | 2.0 | **[1.8; 2.2]** | **<0.0001*** |
| Public | **5.9** | **7.9** | **2.0** | **[1.8; 2.3]** | **<0.0001*** |
| Private | **6.9** | **8.4** | **1.5** | **[0.6; 2.3]** | **0.0010*** |
| Domain 1: Trained provider | 79.7 | 86.4 | 6.7 | [2.3; 11.1] | 0.0030* |
| Public | 80.4 | 86.4 | 6.0 | [1.4; 10.6] | 0.0100* |
| Private | 73.3 | 87.0 | 13.7 | [2.2; -29.5] | 0.0900 |
| Domain 2: Guidelines | 8.1 | 7.5 | -0.6 | [-3.7; 2.5] | 0.6960 |
| Public | 8.7 | 7.2 | -1.5 | [-4.8; -1.8] | 0.3660 |
| Private | 2.2 | 10.8 | 8.6 | [-0.5; -17.6] | 0.0650 |
| Domain 3: Equipment and supplies | 52.1 | 72.6 | 20.5 | [18.4; 22.6] | <0.0001* |
| Public | 50.7 | 72.0 | 21.3 | [19.2; 23.4] | <0.0001* |
| Private | 64.6 | 79.4 | 14.8 | [6.3; 23.4] | 0.0010* |
| Total health facilities | **457** | **804** |  | - | - |
| *Significant at p<0.05. | | | | | |

**Supplemental Table c**

| Table 7: BEmONC service functionality among health facilities providing normal low-risk delivery services in Nepal in 2015 and 2021, by managing authority | | | | | | |
| --- | --- | --- | --- | --- | --- | --- |
| Signal functions | **Managing authority** | **Mean in % (Percentage)** | | **Mean difference in %** | **95% CI of mean difference in %** | **p-value** |
|  |  | **2015** | **2021** |  |  |  |
| 1. All seven signal functions | **Public** | **3.0** | **1.8** | **-1.2** | **[-3.1; 0.8]** | **0.2390** |
|  | **Private** | **15.7** | **10.5** | **-5.2** | **[-18.6; 8.3]** | **0.4470** |
| 1. Administration of parenteral antibiotics | Public | 37.3 | 33.2 | -4.1 | [-9.9; 1.7] | 0.1670 |
|  | Private | 72.3 | 75.2 | 2.9 | [-14.5; 20.3] | 0.7410 |
| 1. Administration of parenteral oxytocin | Public | 86.5 | 88.6 | 2.1 | [1.9; 6.1] | 0.3100 |
|  | Private | 79.0 | 83.1 | 4.1 | [11.5; 19.6] | 0.6080 |
| 1. Administration of parenteral anticonvulsants | Public | 7.7 | 6.1 | -1.6 | [4.7; 1.5] | 0.3160 |
|  | Private | 31.2 | 42.7 | 11.5 | [-7.3; 30.3] | 0.2270 |
| 1. Performed assisted vaginal delivery | Public | 14.1 | 6.1 | -8.00 | [-11.8; -4.2] | <0.0001* |
|  | Private | 34.2 | 32.5 | -1.7 | [-20.3; 16.9] | 0.8550 |
| 1. Performed manual removal of placenta | Public | 41.7 | 35.5 | -6.2 | [-12.1; -0.3] | 0.0390 |
|  | Private | 53.6 | 51.8 | -1.8 | [-21.5; 17.8] | 0.8540 |
| 1. Performed removal of retained products of conception | Public | 31.8 | 24.2 | -7.5 | -13.0; -2.0 | 0.0070 |
|  | Private | 43.9 | 52.1 | 8.2 | [-11.4; 27.8] | 0.4070 |
| 1. Performed neonatal resuscitation | Public | 35.6 | 28.3 | -7.3 | [-12.9; -1.6] | 0.0120* |
|  | Private | 48.0 | 45.2 | -2.8 | [-22.4; 16.9] | 0.7800 |
| Total health facilities |  | **457** | **804** | - | - | - |
| *Significant at p<0.05 | | | | | | |
